# Phytochemical Composition and Antimicrobial and Antioxidant Activity of *Hedysarum semenowii* (Fabaceae)

**DOI:** 10.3390/molecules30234503

**Published:** 2025-11-21

**Authors:** Anel Keleke, Magdalena Maciejewska-Turska, Martyna Kasela, Tomasz Baj, Liliya Ibragimova, Zuriyadda Sakipova, Olga Sermukhamedova, Agnieszka Ludwiczuk

**Affiliations:** 1 Department of Engineering disciplines and Good Practices, Asfendiyarov Kazakh National Medical University, 94 Tole Bi Str., 050012 Almaty, Kazakhstan; ibragimova.l@kaznmu.kz (L.I.); sakipova.z@kaznmu.kz (Z.S.); info@kaznmu.kz (O.S.); 2 Department of Pharmacognosy with the Medicinal Plant Garden, Medical University of Lublin, 1 Chodźki Str., 20-093 Lublin, Poland; magdalena.maciejewska-turska@umlub.edu.pl (M.M.-T.); tomasz.baj@umlub.edu.pl (T.B.); 3 Department of Pharmaceutical Microbiology, Medical University of Lublin, 1 Chodźki Str., 20-093 Lublin, Poland; martyna.kasela@umlub.edu.pl

**Keywords:** *Hedysarum semenowii*, flavones and isoflavones, mangiferin, antioxidant activity, antibacterial activity, antifungal activity

## Abstract

This paper provides a comprehensive phytochemical analysis of extracts obtained from the leaves and roots of *Hedysarum semenowii* using HPLC/PDA-ESI-QToF/MS-MS techniques. The study identified 53 compounds, with flavones and isoflavones as the primary polyphenols. Notably, flavones were predominant in the leaves, while isoflavones were found mainly in the roots, potentially serving as chemotaxonomic markers. Medicarpin and its glucoside were confirmed in the roots, while mangiferin and its derivatives were identified for the first time in both the roots and leaves. Isoflavones like formononetin, calycosin, and afrormosin, along with their glucosides, were exclusive to the roots. Flavonols such as quercetin and its glycosides were abundant in the aboveground parts. Our study also identified flavones like luteolin, flavanones (naringenin), and chalcones (liquiritigenin) in various parts. Additionally, the phenolic acids gallic and ferulic acids, as well as the organic acids malic and citric acid, were also detected. The extracts demonstrated differential antimicrobial and antifungal activities in a microbroth dilution assay, with the aerial part extracts showing superior efficacy, particularly against *Staphylococcus epidermidis* and *Pseudomonas aeruginosa*. Both aerial and underground parts exhibited comparable antifungal activity against *Candida* species. Antioxidant activity in the 1,1-diphenyl-2-picrylhydrazyl (DPPH^•^) radical scavenging test varied significantly, with ethanolic extracts from the aerial parts showing the highest potential (Antioxidant Activity Index (AAI) 2.07 ± 0.13). In contrast, root extracts had consistently low antioxidant activity. The results highlight the aerial parts of *H. semenowii* as a more promising source of biologically active compounds with antimicrobial and antioxidant properties compared to the roots.

## 1. Introduction

*Hedysarum* L. is a genus within the third largest flowering plant family Fabaceae which is distributed across diverse habitats of the Northern Hemisphere, spanning Europe, Asia, North Africa, and North America [1,2].

The genus has significant medicinal importance, with species serving as sources of pharmaceuticals and diagnostic agents [3,4]. Moreover, these plants find utility in fodder cultivation [5,6] and ecological endeavors such as soil enrichment and sand fixation [7,8].

Among the notable species, *Hedysarum polybotrys* Hand.-Mazz., commonly known as “Hong Qi,” stands out for its utilization in traditional Chinese medicine, often as a substitute for the well-established medicinal plant *Astragalus mongholicus* Bunge (Fabaceae) [3,9]. Several pharmacological studies suggest that *H. polybotrys* exhibits pronounced effects compared to *Astragalus mongholicus* [10,11,12], combating aging and associated ailments [3,13], treating gastrointestinal and renal disorders [10,14], aiding postpartum recovery, and enhancing lactation [15,16]. Similarly, *Hedysarum alpinum* L. and *Hedysarum flavescens* Rgl. et Shmalh demonstrate pharmacological versatility, serving as antiviral agents and constituents of sedative, antipyretic, anti-inflammatory, and antimicrobial formulations [17,18,19]. Notably, *Hedysarum neglectum* Ledeb. is recognized for its role in atherosclerosis prevention, treatment of genitourinary system disorders, immune system stimulation, and adaptogenic properties [20,21,22]. *Hedysarum sikkimense* Benth. ex Baker is used in Mongolian traditional medicine to relieve edema of various etiologies [23].

The broad medicinal usage of *Hedysarum* species correlates with their rich chemical composition, encompassing polysaccharides, flavonoids, triterpenoids, coumarins, alkaloids, fatty acids, and other compounds [3,24]. In particular, the antioxidant, anti-aging, antitumor, and antidiabetic properties of *H. polybotrys* are attributed to its polysaccharide content, comprising heteropolysaccharides identified as *Hedysarum polysaccharides 1-4* [14,24]. However, standardization proposals advocate for evaluating the phenolic compound content in *H. polybortys* roots, including calycosin, medicarpin, formononetin, 3-hydroxy-9,10-dimethoxypterocarpan, liquiritigenin, quercetin, formononetin-7-*O*-*β*-D-glucoside, calycosin-7-*O*-*β*-D-glucoside, and genistein, indicating that these compounds are responsible for the pharmacological activity of the plant [25]. *H. alpinum* and *H. flavescens* aerial parts are notable sources of 2-*C*-*β*-D-(glucopyranoside)-1,3,6,7-tetraoxyxanthone, a derivative of mangiferin [26], serving as the active constituent in the antiviral drug Alpizarin^®^ [17,27]. Moreover, these species boast a rich phenolic compound profile, comprising xanthones, flavonols, and hydroxycinnamic and hydroxybenzoic acids: mangiferin, hyperoside, rutin, quercetin 3-L-*α*-rhamnofuranoside, avicularin, hyperoside, polystachoside, hedyserite-1, gallic acid, chlorogenic acid, and others [17,27]. Several *Hedysarum* species serve as alternative sources of mangiferin, including *H. caucasicum* M.Bieb., *H. grandiflorum* Pall., and *H. daghestanicum* Rupr. ex. Boiss [28].

The vast medicinal potential of *Hedysarum* species opens up opportunities to broaden their therapeutic uses. However, historically, taxonomic classification of *Hedysarum* posed challenges owing to uncertainties regarding the genus’s monophyly [1,29]. In view of the repeated taxonomic revisions within the genus [1,29], the study of scientifically recognized species closely related to those widely used in medicine is of particular importance. One such species, *Hedysarum semenowii* Regel & Herder, remains underexplored despite its wide distribution in the Central Tien Shan region across Kazakhstan, Kyrgyzstan, and China’s Xinjiang province [30,31]. *H. semenowii*, belonging to the *Hedysarum* section, shares taxonomic proximity with well-established medicinal species such as *H. polybotrys*, *H. alpinum*, *H. flavescens*, *H. neglectum*, *H. theinum*, and *H. caucasicum* [32,33]. According to the literature, the mentioned species contains compounds such as ononin, formononetin, mangiferin, calycosin, afromosin-7-*O*-*β*-D-glucopyranoside, calycosin-7-*O*-*β*-D-glucopyranoside, afrormosin, medicarpin, hedysarimpterocarpene B, betulinic acid, guanosine, and daucosterol [3,31,32]. Despite the existence of some data on the compounds identified in *H. semenowii*, comprehensive studies on the detailed phytochemical profile of extracts derived from the roots and aerial parts of this plant are still lacking. Moreover, there is insufficient information on the correlation of these findings with data on antimicrobial and antioxidant activities. Therefore, we investigated the chemical composition and antimicrobial and antioxidant activity of *H. semenowii*. These investigations could provide new data for chemotaxonomic differentiation within the genus *Hedysarum* and expand the understanding of the pharmacologically relevant metabolites, as well as revealing the species’ potential for standardization and medicinal use.

## 2. Results

### 2.1. HPLC/PDA-ESI-QToF/MS-MS Analysis

Phytochemical investigations of the underground parts of *Hedysarum* spp. identified flavonoids as the main group of biologically active compounds. Among them, different subclasses have been reported, including flavones, isoflavones, flavanones, xanthones, and pterocarpans [3,34]. Knowledge of the qualitative composition and content of polyphenols present in different parts of *H. semenowii* is relatively poor; to our knowledge there are only two reports published so far [35,36]. Therefore, a detailed phytochemical profile of extracts, obtained from leaves and roots using different extraction solvents and extraction techniques, was conducted. As shown in Table 1, five extracts from the leaves of *H. semenowii* and three extracts from the roots were prepared according to the methods described in Section 4.2.

High-performance liquid chromatography (HPLC/PDA) combined with electrospray ionization quadrupole time-of-flight mass spectrometry (ESI-QToF/MS-MS) enabled the identification of a total of 53 compounds in both the above- and underground parts of the plant. In terms of chemical diversity, flavones and isoflavones were the most representative group of polyphenols detected. Interestingly, their distribution differed significantly; flavones were typical of *H. semenowii* leaves, while isoflavones accumulated mainly in the roots, which could serve as potential chemotaxonomic markers to distinguish between these plant parts. The third group of bioactive constituents commonly found in the *Hedysarum* genus are pterocarpans [3], and the presence of medicarpin and its 3-*O*-glucoside was confirmed exclusively in the subterranean parts of the plant. Additionally, this study reports that the presence of xanthones, namely, mangiferin and its derivatives, were identified in both the roots and leaves of *H. semenowii*, and a detailed analysis of these compounds is presented below. Compounds from each extract were separated chromatographically and subjected to LC-MS analysis in both negative- and positive-ionization mode. Their identification was performed considering retention time and acquired chromatographic (specific UV absorption maxima) and spectrometric data (summarized in Table 2) compared with those reported in the literature and in publicly available MS databases (Human Metabolome Database—HMDB, https://hmdb.ca/) [37].

**Table 2:** Phytochemicals identified in extracts of *Hedysarum semenowi* using HPLC/PDA/ESI-QToF/MS-MS.

No.	Compound	R_t_ (min)	Extract *	Molecular Formula	Exp. (*m*/*z*)	Calcd. (*m*/*z*)	Delta (ppm)	Product Ions (*m*/*z*)	References
1.	Quinic acid	1.98	Hs_M99, Hs_M70, Hs_M50, Hs_Et50M, Hs_Et50U	C_7_H_12_O_6_	191.0557	191.561	2.14	**173.0416**, 127.0364, 111.0409; 85.0273	[37]
2.	Sucrose	1.97	HsR_M99, HsR_M70, HsR_M50	C_15_H_18_O_9_	341.1076	–	–	**179.0549**; 161.0452; 149.0429; 119.0336	[38]
3.	Malic acid	2.14	Hs_M99, Hs_M70, Hs_M50, HsR_M99, HsR_M70, HsR_M50, Hs_Et50M	C_4_H_6_O_5_	133.0138	133.0142	3.33	**115.0029**; 89.0231; 71.0136	[37]
4.	Citric acid	2.62	Hs_M99, Hs_M70, Hs_M50, HsR_M99, HsR_M70, HsR_M50, Hs_Et50M	C_6_H_8_O_7_	191.0175	191.0197	11.59	**111.0085**; 87.0090; 57.0348	[37]
5.	Isocitric acid	4.05	HsR_M99, HsR_M70, HsR_M50	C_6_H_8_O_7_	191.0196	191.0197	0.66	155.009; **111.0075**; 87.0076	[39]
6.	Gallic acid	4.62	Hs_M99, Hs_M70, Hs_M50, Hs_Et50M, Hs_Et50U	C_7_H_6_O_5_	169.0128	169.0142	8.51	**125.0166**; 79.0155; 51.0226	[37]
7.	Hydroxybenzoic acid-*O*-glucoside isomer 1	5.32	Hs_M99, Hs_M70, Hs_M50, HsR_M99, HsR_M70, HsR_M50, Hs_Et50M, Hs_Et50U	C_13_H_16_O_8_	299.0783	299.0772	−3.53	**137.0186**; 93.0300	[37]
8.	Unknown	7.03	Hs_M99, Hs_M70, Hs_M50, HsR_M99, HsR_M70, HsR_M50, Hs_Et50M, Hs_Et50U	-	231.1315	–	–	**213.1220**; 195.1123; 185.1262	
9.	Dihydroxybenzoic acid hexoside	8.23	Hs_M99, Hs_M70, Hs_M50, HsR_M99, HsR_M70, HsR_M50, Hs_Et50M, Hs_Et50U	C_13_H_16_O_5_	315.0710	315.0722	3.66	**153.0271**; 109.0274	[37]
10.	Hydroxybenzoic acid-*O*-glucoside isomer 2	10.77	Hs_M99, Hs_M70, Hs_M50, HsR_M99, HsR_M70, HsR_M50, Hs_Et50M	C_13_H_16_O_8_	299.0745	299.0772	9.13	**137.0129**; 93.0251	[37]
11.	Galloyl glucoside	12.11	Hs_Et50M	C_13_H_16_O_10_	331.0659	331.0671	3.52	313.0541; **169.0096**; 168.0043; 149.9948; 125.0224	[37]
12.	Kaempferol-*C*-glucoside	12.43	HsR_M99, HsR_M70, HsR_M50	C_21_H_22_O_10_	433.1106	433.114	7.88	**343.0830**; 313.0667; 285.0764; 283.0667; 151.0391	fragmentation
13.	Tetrahydroxyxanthone-di-*O*,*C*-hexoside (neomangiferin)	12.89	Hs_M99, Hs_M70, Hs_M50, Hs_Et50M, Hs_Et50U	C_25_H_28_O_16_	583.1284	583.1305	3.52	493.0971; 463.0866; 421.0732; 331.0447; 301.0347; 271.0306; 259.0215	[40]
14.	Mangiferin	14.87	Hs_M99, Hs_M70, Hs_M50, HsR_M99, HsR_M70, HsR_M50, Hs_Et50M, Hs_Et50U	C_19_H_18_O_11_	421.0780	421.0776	−0.86	**331.0394**; 301.0287; 271.0273; 259.0161	[40,41] r.s.
15.	Isomangiferin	16.08	Hs_M99, Hs_M70, Hs_M50, Hs_Et50M, Hs_Et50U	C_19_H_18_O_11_	421.0744	421.0776	7.66	**331.0471**; 301.0366; 259.0259	fragmentation; [41]
16.	Quercetin-*O*-dihexoside	18.89	Hs_M99, Hs_M70, Hs_M50, Hs_Et50M, Hs_Et50U	C_27_H_30_O_17_	625.1393	625.141	2.75	**301.0338**; 300.0279; 271.0217; 255.0315; 179.0028; 151.0007	[42]
17.	Quercetin derivative	19.17	Hs_M99, Hs_M70, Hs_M50, Hs_Et50M, Hs_Et50U	–	755.1970	–	–	**301.0225**; 300.0190; 271.0142; 255.0173; 178.9828; 150.9940	fragmentation
18.	Ferulic acid	19.79	HsR_M99, HsR_M70, HsR_M50	C_10_H_10_O_4_	193.0510	193.0506	−1.89	179.0314; **178.0255**; 149.0607; 134.0367	fragmentation; [42]
19.	Myricetin-*O*-glucoside isomer 1	20.23	Hs_M99, Hs_M70, Hs_M50, Hs_Et50M, Hs_Et50U	C_21_H_20_O_13_	479.0836	479.0831	−1.01	317.0279; **316.0221**; 287.0190; 271.0244; 178.9987; 151.0049	fragmentation
20.	Calycosin-7-*O*-glucoside	20.85	HsR_M99, HsR_M70, HsR_M50	C_22_H_22_O_10_	491.1174 ^*^	491.1195	4.71	**283.0634**; 268.0429; 211.0309; 184.0499	fragmentation
21.	Quercetin-*O*-galactoside	21.16	Hs_M99, Hs_M70, Hs_M50, Hs_Et50M, Hs_Et50U	C_21_H_20_O_12_	463.0897	463.0882	−3.23	**301.0368**; 300.0295; 271.0262; 255.0310; 178.9994; 151.0041	[43,44]
22.	Quercetin-3-glucoside-7-rhamnoside	21.41	Hs_M99, Hs_M70, Hs_M50, Hs_Et50M, Hs_Et50U	C_27_H_30_O_16_	609.1473	609.1461	−1.95	301.0282; **300.0190**; 284.0272; 271.0198; 255.0251; 178.9949; 150.9998	fragmentation
23.	Kaempferol-*O*-dirhamnoside-glucoside	21.68	Hs_M99, Hs_M70, Hs_M50, Hs_Et50M, Hs_Et50U	-	739.1830	–	–	593.1187; **284.0193**; 285.0321; 255.0178; 227.0255; 150.9893	[45]
24.	Quercetin-3-*O*-glucoside	22.46	Hs_M99, Hs_M70, Hs_M50, Hs_Et50M, Hs_Et50U	C_21_H_20_O_12_	463.0897	463.0882	−3.23	403.0546; 343.0350; **301.0266**; 178.9930; 150.9969	[43,44]
25.	Quercetin-3-*O*-arabinoside-7-glucoside	22.94	Hs_Et50M	C_26_H_28_O_16_	595.1278	595.1305	4.46	463.0855; 301.0284; **300.0226**; 271.0184; 179.0165	[46]
26.	Rutoside	23.48	Hs_M99, Hs_M70, Hs_M50, Hs_Et50M, Hs_Et50U	C_27_H_30_O_16_	609.1473	609.1461	−1.95	301.0316; 300.0227; 271.0230; 255.0296; 178.9974; 151.0001	[47,48]
27.	Nicotiflorin	24.18	Hs_M99, Hs_M70, Hs_M50, Hs_Et50M, Hs_Et50U	C_27_H_30_O_15_	593.1500	593.1512	2.01	285.0345; 284.0272; 255.0228; 227.0324; 150.9953	[45]
28.	Hyperoside	24.66	Hs_M99, Hs_M70, Hs_M50, Hs_Et50M, Hs_Et50U	C_21_H_20_O_12_	463.0841	463.0882	8.83	**301.0368**; 300.0295; 271.0262; 255.0310; 178.9994; 151.0041	[49]
29.	Pentahydroxyflavone-*O*-pentoside (Quercetin-*O*-pentoside)	28.27	Hs_M99, Hs_M70, Hs_M50, Hs_Et50M, Hs_Et50U	C_20_H_18_O_11_	433.0862	433.0776	4.23	301.0271; **300.0232**; 271.0219; 255.0246; 243.0297; 178.9921; 151.0034	[46]
30.	Hexahydroxyflavone-*O*-hexoside (Myricetin-*O*-hexoside isomer 2)	28.51	Hs_M99, Hs_M70, Hs_M50, Hs_Et50M, Hs_Et50U	C_21_H_20_O_13_	479.0808	479.0831	4.82	**317.0135**; 316.0078; 178.9947; 151.0001; 137.0207	fragmentation
31.	Tetrahydroxyflavone- 3-*O*-hexoside (Kaempferol-*O*- hexoside)	29.17	Hs_M99, Hs_M70, Hs_M50, Hs_Et50M, Hs_Et50U	C_21_H_20_O_11_	447.0933	447.0933	−0.03	285.0364; **284.0328**; 255.0289; 151.00	[45,47]
32.	Tetrahydroxyflavone-3-*O*-hexoside-pentoside (Kaempferol-3-*O*- hexoside-pentoside) isomer 2	29.44	Hs_Et50M	C_27_H_30_O_15_	593.1474	593.1512	6.39	**285.0345**; 284.0272; 255.0354; 151.0004	[45,47]
33.	Isorhamnetin-3-*O*- hexoside	30.07	Hs_Et50M, Hs_Et50U	C_22_H_22_O_12_	477.1033	477.1038	1.15	447.0863; **315.0404**; 314.0410; 271.0492; 151.0006	fragmentation
34.	Quercetin-3-*O*- malonylglucoside	31.75	Hs_Et50M, Hs_Et50U	C_24_H_22_O_15_	549.0917	549.0886	−5.65	505.0982; 463.0928; **301.0329**; 300.0272; 271.0245; 255.0308; 179.0063; 151.0041	[45]
35.	Pseudobaptigenin-*O*- glucoside	32.93	HsR_M99, HsR_M70, HsR_M50	C_22_H_20_O_10_	489.1021 ^*^	489.1038	3.94	**281.0468**; 253.0516; 225.0576; 135.0144	[49]
36.	Ononin	34.40	HsR_M99, HsR_M70, HsR_M50	C_22_H_22_O_9_	475.1259 *	475.1246	−3.06	**267.0630**; 252.0438	[48]
37.	Afrormosin-*O*-glucoside (wistin)	36.29	HsR_M99, HsR_M70, HsR_M50	C_23_H_24_O_10_	505.1339 ^*^	505.1351	2.72	**297.0775**; 282.0533; 267.0293; 254.0803; 239.0333; 195.0482	[50]
38.	Quercetin-*O*-acetyl- glucoside	36.57	Hs_Et50M, Hs_Et50U	C_23_H_22_O_13_	505.1002	505.0988	2.84	445.0757; 427.0680; 343.0412; 301.0327	fragmentation; [49]
39.	Liquiritigenin	38.14	HsR_M99, HsR_M70, HsR_M50	C_15_H_12_O_4_	255.0656	255.0663	−2.66	**135.0086**; 119.0492	[42]
40.	Calycosin	40.62	Hs_M99, Hs_M70, Hs_M50, HsR_M99, HsR_M70, HsR_M50, Hs_Et50M, Hs_Et50U	C_16_H_12_O_5_	283.0606	283.0612	2.1	**268.0387**; 224.0413; 211.00402; 195.0403; 184.0519; 148.0168; 135.0100; 120.0214	[49]
41.	Medicarpin-3-*O*- glucoside	40.86	HsR_M99, HsR_M70, HsR_M50	C_22_H_24_O_9_	477.1411	477.1402	−1.81	431.1238; **269.0807**; 254.0481; 161.0228; 133.0298; 132.0207	[51]
42.	Quercetin	42.78	Hs_M99, Hs_M70, Hs_M50, Hs_Et50M, Hs_Et50U	C_15_H_10_O_7_	301.0362	301.0354	−2.73	273.0402; 257.0402; 229.0485; 178.9985; **151.0026**; 149.0323; 121.0290; 107.0137	[52,53]
43.	Luteolin	43.11	Hs_Et50M, Hs_Et50U	C_15_H_10_O_6_	285.0428	285.0405	−8.17	151.0004; **133.0224**	[37,54]
44.	Isoformononetin	43.30	HsR_M99, HsR_M70, HsR_M50	C_16_H_12_O_4_	267.0659	267.0663	1.43	**252.0393**; 223.0393; 208.0530; 195.0461; 135.0315; 132.0214	[55]
45.	Naringenin	45.16	Hs_M99, Hs_M70, Hs_M50, HsR_M99, HsR_M70, HsR_M50, Hs_Et50M, Hs_Et50U	C_15_H_12_O_5_	271.0616	271.0612	−1.48	227.0837; 177.0212; **151.0034**; 119.0478; 107.0177; 93.0314	[42]
46.	Sissotrin-4′-*O*-glucoside	42.84	HsR_M99, HsR_M70, HsR_M50	C_22_H_22_O_10_	491.1209 ^*^	491.1195	−3.14	445.1154; **283.0598**; 268.0292	[49]
47.	Pseudobaptigenin	50.06	Hs_M99, Hs_M70, Hs_M50, HsR_M99, HsR_M70, HsR_M50, Hs_Et50M, Hs_Et50U	C_16_H_10_O_5_	281.0462	281.0455	−2.32	**253.0499**; 251.0360; 223.0396; 195.0449; 135.0093	[49,56]
48.	Formononetin	50.95	Hs_M99, Hs_M70, Hs_M50, HsR_M99, HsR_M70, HsR_M50, Hs_Et50M, Hs_Et50U	C_16_H_12_O_4_	267.0654	267.0663	3.29	**252.0389**; 223.0407; 208.0541; 195.0457; 135.0251; 132.0215	[49]
49.	Afrormosin	51.37	HsR_M99, HsR_M70, HsR_M50	C_17_H_14_O_5_	297.0743	297.0768	8.54	**282.0533**; 267.0293; 254.0803; 239.0333; 195.0482	fragmentation
50.	Isoliquiritigenin	52.03	Hs_M99, Hs_M70, Hs_M50, HsR_M99, HsR_M70, HsR_M50, Hs_Et50M, Hs_Et50U	C_15_H_12_O_4_	255.0656	255.0663	2.66	**135.0081**; 119.0466; 91.0184	[42]
51.	Irilone	52.62	HsR_M99, HsR_M70, HsR_M50	C_16_H_10_O_6_	297.0385	297.0405	−6.58	282.438; **269.0455**; 267.0289; 241.0502;	fragmentation; [56]
52.	Medicarpin	54.13	HsR_M99, HsR_M70, HsR_M50	C_16_H_14_O_4_	269.0790	269.0819	10.86	**254.0598**; 161.0279; 145.0306; 133.0288; 132.0223	[51]
53.	Biochanin A	59.06	HsR_M99, HsR_M70, HsR_M50	C_16_H_12_O_5_	283.0637	283.0612	−8.81	**268.0381**; 240.0419; 239.0377; 211.0410; 224.0489; 240.0419; 195.0441; 183.0440; 163.0036; 151.0054; 135.0097; 132.0208; 109.0303	[49]

* Hs_M99—*H. semenowii* leaf extract in methanol 99.8%, Hs_M70—*H. semenowii* leaf extract in methanol 70%, Hs_M50—*H. semenowii* leaf extract in methanol 50%, Hs_Et50M—*H. semenowii* leaf extract in ethanol 50% (maceration), Hs_Et50U—*H. semenowii* leaf extract in ethanol 50%50% (ultrasonification), HsR_M99—*H. semenowii* root extract in methanol 99.8%, HsR_M70—*H. semenowii* root extract in methanol 70%, HsR_M50—*H. semenowii* root extract in methanol 50%, ^*^—[M + HCOO]^−^, r.s.—identity of the compound additionally confirmed using a reference standard.

#### 2.1.1. Isoflavones

Isoflavonoids are specialized metabolites characterized by biological activity resembling that of the endogenous hormone 17-β-oestradiol. These compounds have been previously detected in underground parts of several *Hedysarum* spp. Our results are in accordance with data reported in the literature, as the presence of formononetin (**48**), calycosin (**40**), afrormosin (**49**) and their corresponding *O*-glucosides, ononin (**36**), calycosin-7-*O*-glucoside (**20**) and wistin (**37**), has been confirmed exclusively in *H. semenowii* roots [3,34]. Aglycones of isoflavones were characterized by the successive loss of small moieties from the deprotonated [M-H]^−^ ions. The identification of *retro*-Diels-Alder (rDA) reaction products (rDA product ions) provided information on the number and type of substituents attached to the aromatic rings [57]. The precursor ions of isoflavone *O*-glucosides were observed as solvent adducts [M+HCOO]^−^. Cleavage of the O-C bond (−162 Da) in their structure resulted in the formation of an intense peak in the MS/MS spectra, corresponding to the free aglycone. The elution order of chromatographically separated isoflavones was similar to that previously described by Maciejewska-Turska and Zgórka (2022) [49]. Among the identified isoflavones, formononetin (*m*/*z* 267.0654) and ononin (adduct ion at *m*/*z* 475.1259) were the major compounds. Minor peaks for biochanin A (**53**), pseudobaptigenin (**47**), and their corresponding glucosides, sissotrin-4′-*O*-glucoside (**46**) and pseudobaptigenin-*O*-glucoside (**35**), were identified based on our experience and information obtained from online MS databases [37]. Two compounds (**49** and **51**) had a similar [M-H]^−^ deprotonated molecular ion at *m*/*z* 297. Compound **49**, eluting at 51.37 min, generated a precursor ion at *m*/*z* 297.0743, consistent with the molecular formula of C_17_H_14_O_5_. The MS/MS spectrum of this compound contained abundant product ions at *m*/*z* 282.0533 and 267.0293, which matched the fragmentation pattern typical of methoxylated isoflavones (−15 Da). Among known isoflavones bearing two methoxyl groups, previously reported in the genus *Hedysarum*, afrormosin was proposed as the most likely structure [3]. For compound **51**, the product ion at *m*/*z* 297.0385 suggested a molecular formula of C_16_H_10_O_6._ An intense product ion at *m*/*z* 269.0455 corresponded to the loss of 30 Da, prevalent among methylenedioxy-substituted isoflavones, such as pseudobaptigenin or irilone [56].

Therefore, compound **49** was assigned as afrormosin, whilst compound **51**, with a later elution, could be assigned as irilone. For compound **44**, a similar fragmentation pattern to formononetin was observed, and consequently it was proposed to be isoformononentin.

#### 2.1.2. Flavonols, Flavones, and Flavanones

The flavonols identified in the extracts obtained from leaves of *H. semenowii* were represented mainly by quercetin and less abundantly by kaempferol derivatives (Table 2). Small shifts in their absorption maxima, together with diagnostic fragmentation ions observed in their MS/MS spectra at *m*/*z* 301 and 285, suggested that the aglycone cores originated from quercetin or kaempferol, respectively [45]. Product ions (^1.3^A^−^ and/or ^1.3^B^−^) arising from rDA cyclization were found to be the most useful for flavonoid identification. Quercetin (**42**) and eleven of its glycosides (**16**, **17**, **21**, **22**, **24**, **25**, **29**, **34,** and **38**) were confirmed to be present. The MS spectrum of compound **42** at *m*/*z* 301.0362 (C_15_H_10_O_7_) and a series of fragmentation ions at *m*/*z* 273.0402 [M-H-CO]^−^, 257.0402 [M-H-CO_2_]^−^, and 229.0485 [M-H-CO-CO_2_]^−^, together with rDA ions at *m*/*z* 178.9985 (^1.2^A^−^), 151.0026 (^1.3^A^−^), 121.0290 (^1.2^B^−^), and 107.0137 (^0.4^A^−^), supported the quercetin structure [52,53]. A key step in the identification of quercetin glycosides was the observation of neutral losses of 162 Da or 146 Da, indicating the presence of glucose or rhamnose substituents, respectively. For these compounds, the aglycone was recorded at *m*/*z* 301.03, while similar fragmentation ions in their MS/MS spectra to those described for quercetin confirmed the quercetin core [46,52]. As a result, quercetin-*O*-dihexoside (**16**), quercetin derivative (**17**), quercetin-3-glucoside-7-rhamnoside (**22**), quercetin-3-*O*-arabinoside-7-glucoside (**25**), rutoside (**26**), hyperoside (**28**), and quercetin-*O*-pentoside (**29)** were tentatively identified [45]. Since the use of spectrometric (MS) techniques alone does not provide information on the specific type of hexose substituent, the elution behavior and available literature data on the flavonoid profile of several *Hedysarum* species allowed the preliminary identification of compound **21** as quercetin-3-*O*-galactoside, and compound **24** was eluted later as quercetin-3-*O*-glucoside, previously noted in methanolic extracts of *Hedysarum vicioides* leaves [43,44,45]. In addition, one quercetin-3-*O*-malonylglucoside (**34**) and one quercetin-*O*-acetyl-glucoside (**38**) were also identified in the aboveground parts of the plant, based on the characteristic losses of malonyl (−86 Da and −44 Da) and acetyl (−42 Da) moieties [45]. Compounds **12**, **23**, **27**, **31,** and **32** were tentatively identified as kaempferol glycosides, namely, kaempferol-*C*-glucoside, kaempferol-*O*-dirhamnoside-glucoside, nicotiflorin, kaempferol-*O*-hexoside, and kaempferol-3-*O*-hexoside-pentoside (an isomer of nicotiflorin), respectively [45,47]. Diagnostic fragment ions observed in their MS/MS spectra at *m*/*z* 285.0667 and 151.0391 (^1.3^A^−^) indicated that these compounds originated from kaempferol rather than luteolin. In terms of compound **12**, the generated precursor ion at *m*/*z* 433.1106 was in accordance with the empirical molecular formula of C_21_H_22_O_10_. The most intensive product ions at *m*/*z* 343.0830 [M-H-90]^−^ and 313.0667 [M-H-120]^−^ confirmed that the hexose unit was attached to the aglycone core via a C-C bond and underwent cross-ring cleavage, characteristic of *C*-glycosides [54]. Two isomers of myricetin-*O*-glucoside (**19**, **30**) and one peak identified as isorhamnetin-3-*O*-hexoside (**33**) were also noted. Luteolin (**43**) was detected solely in ethanolic (50%, *v*/*v*) extracts of the aboveground parts of *H. semenowii.* In MS/MS spectra, a precursor ion observed at *m*/*z* 285.0428 and two distinctive rDA product ions at *m*/*z* 151.0004 and 133.0224, obtained through 10-eV collision-induced dissociation (CID), confirmed the structure of luteolin [54]. In addition, one flavanone, namely, naringenin (**45**), was identified by comparing our recorded elution behavior and UV-vis and MS spectra with information available in the literature [42].

#### 2.1.3. Chalcones

According to the LC/MS results, it was proposed that two compounds (**39** and **50**) were chalcone-type natural products. Compound **39**, detected solely in root extracts, yielded a deprotonated molecular ion at *m*/*z* 255.0656 (C_15_H_12_O_4_), which upon further fragmentation produced two major fragment ions at *m*/*z* 135.0086 and 119.0492. Compound **50** shared a similar fragmentation pathway to that of compound **39**, consistent with publicly available MS data reported for liquiritigenin and isoliquiritigenin [42,58]. Based on their retention behavior, compound **39**, eluting at 38.14 min, was assigned as liquiritigenin, while compound **50**, identified in all analyzed samples, was assigned as isoliquiritigenin. These findings are in agreement with previously reported data for several *Hedysarum* spp. [3].

#### 2.1.4. Pterocarpans

Compound **52**, due to its apolar structure, eluted at 54.13 min and yielded a dominant [M-H]^−^ precursor ion at *m*/*z* 269.0775, which was in accordance with the empirical molecular formula C_16_H_14_O_4_. An intense fragment ion at *m*/*z* 254.0598 suggested the loss of one CH_3_ group. Further fragmentation corresponded well with data reported for medicarpin, previously identified in *Coffea* leaf extracts by Wang et al. (2019) [51] and in several *Hedysarum* species [34]. These authors suggested that the product ions observed in MS/MS spectra may correspond to the cleavage of 6.8 (*m*/*z* 161.0279) 6.7 (*m*/*z* 145.0306), and 3.4.7 (*m*/*z* 133.0288) bonds in the A-ring and a 3.5 bond in the D-ring (*m*/*z* 132.0223) of medicarpin. Based on these data and the MS spectrum of compound **52**, detected exclusively in roots, it was assigned as medicarpin. Similar fragmentation behavior was observed for compound **41**. The mass difference between the adduct ion [M+HCOO]^−^ at *m*/*z* 477.1367 (C_22_H_24_O_9_) and the predominant product ion at *m*/*z* 269.0807 resulted from the typical neutral loss of one hexose moiety. Moreover, the experimentally determined accurate mass of the fragment ion at *m*/*z* 269.0807 was in good agreement with the proposed molecular formula of C_16_H_14_O_4_, corresponding to the medicarpin core rather than a trihydroxyflavone structure. Therefore compound **41** was assigned as medicarpin-3-*O*-glucoside.

#### 2.1.5. Xanthones

Regarding xanthones, three specific high-intensity absorption maxima in the range of 250–370 nm were observed [59]. Compound identification was performed by comparing fragmentation ions formed after CID with the literature MS data. Compound **14** yielded a deprotonated ion [M-H]^−^ at *m*/*z* 421.0828, which was consistent with the empirical molecular formula of C_19_H_18_O_11_. The most abundant ions at *m*/*z* 331.0471 and 301.0366 were formed due to the losses of 120 Da and 90 Da, respectively, from the precursor ion through cross-ring cleavage of the sugar moiety [48]. It was therefore postulated that the substitution of one hexose moiety was via a C-C bond. Further fragmentation resulted in fragment ions consistent with data published by Trevisan et al. [60] for a specific *C*-glycosylated xanthone, namely, mangiferin, registered under the name Alpizarin^®®^ in the Russian Federation [41]. Comparison of spectroscopic and spectrometric data with the MG, reference standard, enabled the identification ofcompound **14** as mangiferin. To our knowledge, this is the first study to report the presence of mangiferin in both *H. semenowii* aerial and underground organs. Compounds **13** and **15**, observed exclusively in the aerial parts of *H. semenowii*, shared similar fragmentation behavior to mangiferin. The lack of prominent product ion at *m*/*z* 271 in the MS/MS spectra of compound **15** suggested a structural isomer of mangiferin, tentatively identified as isomangiferin [61]. Regarding compound **13**, the mass difference between the precursor ion [M-H]^−^ at *m*/*z* 583.1284 (C_25_H_28_O_16_) and two major fragmentation ions at *m*/*z* 493.0971 [M-H-90]^−^ and 463.0866 [M-H-120]^−^ supported the presence of a sugar at the C-2 position, whilst a product ion at *m*/*z* 421.0732 [M-H-162]^−^ could be attributed to the cleavage of an *O*-glucosidic bond. Less abundant product ions typical of mangiferin were also detected. According to the literature data, compound **13** could correspond to neomangiferin, a naturally occurring xanthone derivative with antidiabetic activity, bearing both *O*- and *C*-glucosidic residues [40]. However, data obtained solely from MS/MS spectra were insufficient to unambiguously determine the position of the glycosylation site, so compound **13** was tentatively assigned as a tetrahydroxyxanthone-di-*O*, *C*-hexoside [61].

#### 2.1.6. Phenolic Acids and Other Compounds

Out of the six phenolic acids identified in *H. semenowii,* one was dihydroxybenzoic acid hexoside (**9**) and two were isomers of hydroxybenzoic acid-*O*-glucoside (**7** and **10**). Ferulic acid (**18**) was identified exclusively in the roots, while gallic acid (**6**) and its glucoside (**11**) were detected only in the aerial parts.

In addition, several organic acids, such as malic (**3**) and citric acid (**4**), were present in all samples. Quinic acid (**1**), with a precursor ion at *m*/*z* 191.0513, was noticed in the aboveground parts, whilst isocitric acid (**5**) with an [M-H]^−^ at *m*/*z* 191.0196 was detected solely in the roots [39].

### 2.2. Quantification of Mangiferin and Isomangiferin via RP-LC/PDA

Preliminary RP-LC/PDA analysis revealed that mangiferin was the dominant compound in extracts obtained from the aerial parts of *Hedysarum semenowii*. For mangiferin determination, the aforementioned *H. semenowii* samples were injected in triplicate and the results are presented in Table 3 as the mean of three independent analyses, expressed as the quantity per 1 g of the extracts [mg/g] ± standard deviation with relative standard deviation. Quantitative analysis showed considerable variation in mangiferin content among the extracts, with almost three-fold higher concentration in the ethanolic (50%, *v*/*v*) extract compared to the others (Figure S1). Comparison of the mangiferin concentrations in samples Hs_M99, Hs_M70, and Hs_M50 indicated that the alcohol concentration used during the extraction process did not significantly affect the amount of mangiferin detected. The content of isomangiferin was calculated using the calibration curves of mangiferin, due to the similarity of their UV-vis spectra and chromatographic behavior. Isomangiferin levels were relatively low and, like with mangiferin, were highest in ethanolic extracts. The amount of neomangiferin was undetectable in all samples.

### 2.3. Antibacterial and Antifungal Activity

The present study evaluated the antimicrobial and antifungal potential of extracts of *Hedysarum semenowii* (Table 4 and Table 5). Aerial part extracts demonstrated higher antibacterial activity compared to root extracts, as evidenced by lower Minimum Inhibitory Concentration (MIC) and Minimum Bactericidal Concentration (MBC) values against various microorganisms.

Against Gram-positive reference bacteria from the *Staphylococcus* genus, extracts from *H. semenowii* leaves exhibited notable activity, with MIC values ranging from 0.5 to 4 mg/mL. Particularly, a significant inhibitory effect was observed against *S. epidermidis* ATCC 12228. Additionally, leaf extracts displayed considerable activity against the Gram-negative *P. aeruginosa* ATCC 27853 (MIC = 1–2 mg/mL), notably higher than for *E. coli* ATCC 25922 (MIC = 8 mg/mL).

Among the tested samples, root extracts exhibited noteworthy activity against *S. epidermidis* ATCC 12228; interestingly, the activity increased proportionally with the concentration of methanol used as the extraction solvent. However, their efficacy against other bacterial strains was comparatively lower, requiring MIC ≥ 4 mg/mL for effective inhibition.

In terms of antifungal activity against *Candida* species, both underground and aboveground part extracts demonstrated comparable efficacy, with MIC values ranging from 4 to 16 mg/mL.

Calculated MBC/MIC and MFC/MIC ratios indicated that extracts demonstrated bactericidal and fungicidal properties.

These findings underscore the differential antimicrobial and antifungal properties exhibited by various parts of *H. semenowii*, with the leaves demonstrating superior efficacy against bacterial pathogens, while both underground and aboveground parts displayed comparable activity against fungal strains.

Statistical analysis showed that the studied *H. semenowii* extracts varied significantly in terms of their activity (MIC values) towards certain bacterial reference strains; however, these differences were not observed in the case of antifungal activity (Table 6). Despite significant differences, post hoc pairwise comparisons only allowed us to identify specific group differences in antimicrobial efficacy in two species. The analysis showed that these differences were visible for antistaphylococcal activity of Hs_M99 and HsR_M50 extracts against *S. aureus* ATCC 29213, as well as for activity against *B. cereus* ATCC 10876 between Hs_Et50M and HsR_M50.

### 2.4. Antioxidant Activity

The antioxidant activity of the extracts of *H. semenowii* was evaluated, and notable differences in antioxidant potential between the leaves and root extracts were observed.

Extracts from the aerial part (leaves) displayed varying degrees of antioxidant activity (Table 7). Notably, ethanolic extracts (Hs_Et50M and Hs_Et50U) showcased strong antioxidant potential, with Antioxidant Activity Index (AAI) values of 2.07 ± 0.13 and 1.49 ± 0.26, respectively. Extracts obtained with methanol exhibited different levels of activity, as Hs_M99 showed strong antioxidant activity with an AAI of 1.6 ± 0.2, while Hs_M70 demonstrated moderate activity with an AAI of 0.90 ± 0.09. Conversely, Hs_M50 displayed poor antioxidant activity, as indicated by an AAI of 0.37 ± 0.05.

In contrast, extracts derived from *H. semenowii* roots exhibited consistently low antioxidant activity across all samples during initial screening. Thus, these results were not included in this work. These findings underscore the differential antioxidant capabilities of *H. semenowii* extracts, with leaf extracts displaying a spectrum of antioxidant activity, particularly high in ethanolic extracts, while root extracts showed uniformly low antioxidant potential.

## 3. Discussion

Phytochemical analysis of *Hedysarum semenowii* extracts revealed a range of phytochemicals, consisting predominantly of flavones, isoflavones, xanthones, and pterocarpans. These results mainly support previously published studies on this plant and its closely related species, as well as reports indicating that extraction using moderately polar solvents (50–70% methanol or ethanol) provides optimal yields of biologically active constituents [3,32,48].

The *H. semenowii* leaf extracts were rich in flavonols and xanthones, such as quercetin, kaempferol, mangiferin, and their derivatives. Flavonols are a major subclass of polyphenols known for their antioxidant activity, primarily due to the presence of phenolic hydroxyl groups capable of donating hydrogen atoms to free radicals [62]. Quercetin, kaempferol, luteolin, and their glycosides identified in the analyzed extracts are known to have significant biological activities, particularly against diseases and conditions related to aging, inflammations, tumors, and oxidative stress [21,63,64,65]. Quercetin and its derivatives have displayed significant antioxidant activity compared to butylated hydroxytoluene and *Allium cepa* extract in DPPH^•^ scavenging and ferric reducing ability in plasma (FRAP) tests [66]. Similarly, kaempferol and its derivatives, as well as plant extracts containing these compounds, have shown high antioxidant activity in DPPH^•^, superoxide radical, and nitric oxide (NO) inhibition assays, with results comparable to those of ascorbic acid [67,68]. The ability of these compounds to counteract oxidative stress plays a key role in their therapeutic potential against autoimmune, cardiovascular, and certain neurological diseases, as well as various cancer types [67].

Mangiferin and its derivatives, previously reported to be present only in the aerial parts of *Hedyrasum* L. species [31], were also detected in the roots of *H. semenowii*, although in significantly lower quantities compared to the leaves. The antiviral activity of mangiferin and isomangiferin is well-documented [17,27]. Some studies report their efficacy to be greater than that of acyclovir, idoxuridine, and cyclocytidine against herpes simplex virus, with isomangiferin demonstrating superior activity [69]. In addition to their antiviral properties, mangiferin and its derivatives have shown moderate to mild antimicrobial activity against strains such as *Staphylococcus aureus*, *Escherichia coli*, *Candida albicans*, and *Aspergillus niger*, as evidenced by disk diffusion assays [70]. Furthermore, these compounds are known for a broad spectrum of pharmacological effects, including antioxidant, antidiabetic, anti-inflammatory, and anticancer activities [71].

According to this study, the identified isoflavones, pterocarpans, and chalcones were found exclusively in the root extracts of *H. semenowii*. Isoflavones such as formononetin, calycosin, and afrormosin are known phytoestrogens, typically present in subterranean parts of plants belonging to the *Fabaceae* family, with *Astragalus membranaceus* being a primary source [72,73]. Their biological activity is associated with binding to estrogen receptors, which contribute to their potential effects against various cancer types, as well as their anti-inflammatory, neuroprotective, and hepatoprotective properties [72,73]. Medicarpin, a pterocarpan, has been reported to exhibit anti-osteoporotic activity by suppressing osteoclastogenesis in bone marrow cells [74]. It also shows antifungal activity against *Trametes versicolor* and antigonococcal activity against *Neisseria gonorrhoeae* [75,76] and neuroprotective effects [77]. Chalcones identified in the root extracts, such as liquiritigenin and isoliquiritigenin, are known to exhibit hepatoprotective and anti-inflammatory effects, the latter one of which is primarily attributed to their ability to reduce the production of inducible nitric oxide synthase (iNOS) and proinflammatory cytokines [78,79].

Notably, this study reports for the first time the presence of ferulic, malic, quinic, citric, and isocitric acids in a species of *Hedysarum* L., specifically, *H. semenowii* [3]. These compounds, commonly found in various medicinal and edible plants, are known for their antioxidant, anti-inflammatory, and metabolism-regulating properties. For instance, ferulic acid has demonstrated strong radical-scavenging activity and is associated with cardiovascular and neuroprotective effects [80,81,82]. Organic acids such as malic and quinic acids have also been linked to antioxidant and antimicrobial activities [83,84]. Similarly, citric and isocitric acids are well-known for their antioxidant potential, with citric acid widely used in the pharmaceutical industry as a metal-chelating and pH-regulating agent [85,86]. The detection of these organic acids reveals a broader phytochemical profile in *H. semenowii* and plants of *Hedysarum* L., as well as highlighting the potential of this underexplored species as a valuable source of bioactive compounds.

However, despite literature reports documenting the presence of compounds such as hedysarimpterocarpene B, betulinic acid, guanosine, and daucosterol in *H. semenowii* [3], these compounds were not detected in the analyzed extracts. Their absence may be attributed to several factors, including differences in the collection region or vegetative stage, the extraction methodology used, the concentration of the compounds, or variations in sample preparation and taxonomic identification [1,29]. This study was conducted using a single population sample of *H. semenowii*, which represents a limitation, as phytochemical composition may vary among different ecological sources.

When analyzing the antimicrobial and antioxidant activities of *H. semenowii* extracts, it was found that the leaf extracts exhibited more pronounced effects compared to those obtained from the roots. These effects align with the known properties of the compounds identified in the extracts [21]. The root extracts of *H. semenowii* contained isoflavonoids, pterocarpans, and chalcones, which, according to published data, are not typically associated with strong antimicrobial or antioxidant properties. Instead, they are more recognized as phytoestrogens and anti-osteoporotic agents, with potential applications in the treatment of inflammatory conditions and chronic diseases such as cancer [3,34,72]. Although some studies have reported on medicarpin’s antimicrobial activity, its efficacy is considered relatively weak in comparison to flavonols [75].

The results of this study emphasize that the biologically active phenolic profile of *H. semenowii* is more concentrated in the aerial parts, which demonstrated superior performance in antioxidant and antimicrobial assays. Even though the biological activity assays used in this study were based on simplified conventional methods and do not reflect the complexity of in vivo activity mechanisms, they provide a useful preliminary screening of the comparative activity of extracts from different parts of the plant. This suggests that aboveground biomass of the plant, being more accessible for harvesting, may serve as a promising natural source of bioactive compounds with these properties. Conversely, the roots, as a source of phytoestrogens, may be better suited to potential therapeutic applications related to hormone modulation.

Given the phytochemical similarities between *H. semenowii* and other well-studied *Hedysarum* species such as *H. alpinum*, *H. flavencens*, and *H. polybortys*, these findings support the idea that this underexplored species holds significant pharmacological potential. Future research should prioritize the isolation of individual compounds for activity-guided studies, assessment of synergistic interactions, and in vivo evaluation of therapeutic safety and efficacy.

## 4. Materials and Methods

### 4.1. Plant Material

The leaves of *Hedysarum semenowii* Regel & Herder were collected during the flowering period in July 2023 from mountainous areas of the Kungey Alatau ridge, Kegen district, Almaty region, Republic of Kazakhstan (coordinates: N 43°00′18″, E 78°22′58.6″, elevation 1740 m), when aboveground parts had reached maximum biomass and metabolite content. Subsequently, root specimens of *H. semenowii* were collected in the same areas in September 2023, following the conclusion of the flowering period and accumulation of secondary metabolites later in vegetation. The collected samples underwent identification by specialists from the Republican State Enterprise with the Right of Economic Management (RSE REM) “Institute of Botany and Phytointroduction”, which operates under the Forestry and Wildlife Committee Ministry of Ecology, Geology and Natural Resources of the Republic of Kazakhstan. Identification was conducted using morphological characteristics and other relevant methods.

### 4.2. Extraction

Eight extracts of *H. semenowii* were prepared from both leaves and roots. Aerial parts were finely chopped and subjected to an extraction process using anhydrous methanol, 70% methanol, and 50% methanol (*v*/*v*) through ultrasonic maceration. The maceration was performed at room temperature with three 30 min cycles of ultrasonic treatments each time, using a fresh portion of the solvent at a ratio of 1:10 (weight/volume). All extracts were concentrated to dryness using a rotary evaporator at a temperature not exceeding 50 °C. Similarly, *H. semenowii* root extracts were prepared following the same procedure.

In addition, two types of aerial part extracts were prepared. The first extract was obtained through maceration using 50% ethanol at room temperature, while the second extract additionally underwent ultrasonic treatment in three short 5 min pulses. Both extracts were subsequently freeze-dried to dryness. The abovementioned solvents were chosen to represent different levels of polarity, enabling the extraction of a broad range of constituents, as well as the comparison of extraction efficiency and solvent selectivity.

### 4.3. LC and LC-MS Analysis

#### 4.3.1. Chemical Reagents

HPLC-grade solvents (acetonitrile and formic acid) were supplied by Sigma Aldrich (Steinheim, Germany). Purified water (18.2 MΩ) was prepared by the Direct-Q system (Millipore, Molsheim, France). Solvents of spectroscopic purity, used for LC/PDA/ESI-QToF/MS-MS analysis (acetonitrile, formic acid, and water), were provided by J.T. Baker (Gross-Gerau, Germany). All other chemicals were of analytical reagent grade. The reference standard of mangiferin (MG) used for qualitative analysis had purity greater than 93% and was purchased from ChromaDex Inc. (Santa Ana, CA, USA). A stock solution of mangiferin was prepared by dissolving the standard in 50% methanol (HPLC-grade) to obtain a concentration of 0.2 mg/mL.

#### 4.3.2. RP-LC/PDA Profiling

Extracts were analyzed both quantitatively and qualitatively using an Agilent 1100 (Agilent Technologies, Inc., Santa Clara, CA, USA) chromatograph equipped with an autosampler, a photodiode-array detector (DAD), and a thermostatic column. Separation of polyphenols was carried out on a Zorbax Eclipse XDB-C18 column (250 × 4.6 mm I.D., d_p_ = 5 μm) at 25 °C. An optimized gradient of the mobile phase, consisting of acetonitrile (B) (*v*/*v*) and water (A), with the addition of 0.1% formic acid to both eluents, was used as follows: 0/10; 13/25; 15/95; isocratic run at 95% from 15 to 20 min and back to 10% in 25 min/%B in A. The flow rate was set to 1 mL/min, and a sample injection volume of 10 μL was used. The identification of compounds was based on their specific UV spectra recorded at 254 nm and retention time. The concentration of mangiferin (MG) in extracts from *Hedysarium semenowii* leaves was quantitatively determined using the external standard method. The content of its structural isomer, isomangiferin, was calculated as mangiferin, due to their nearly identical UV-vis spectra and chromatographic behavior. For this purpose, a set of methanolic solutions of MG was prepared in triplicate at different concentrations, ranging from 0.0125 to 0.2 mg/mL. A five-point calibration curve was constructed (peak areas versus increasing concentrations of reference solution). The calibration curves obtained were highly linear, with a correlation coefficient (R^2^) value of 0.9993 within the test ranges.

Regarding the method precision, the standard solution of MG was prepared in triplicate at a concentration of 0.0125 mg/mL. The prepared solutions were injected on the same day and on three consecutive days of the study for the determination of intra- and interday precision. The precision, expressed as the relative standard deviation (RSD, %) was below 2%, which confirmed the high stability of the method (Table 8). The lowest concentration of the MG standard was diluted with methanol and injected at a volume of 10 µL to determine the limit of detection (LOD) and limit of quantification (LOQ). Under the optimized chromatographic conditions, the LOD was defined as a signal-to-noise ratio (S/N) of 3:1 and the latter as an S/N of 10:1.

#### 4.3.3. RP-LC/DAD/ESI–QToF/MS/MS Analysis

Extracts were subjected to LC-MS analysis performed on an Agilent 1260 Infinity chromatograph connected to a 6530B Accurate Mass QTOF/MS mass spectrometer (Agilent Technologies, Santa Clara, CA, USA). As a soft ionization technique, the ESI ion source operated in both positive- and negative-ionization modes. The chromatographic separation was attained on a Zorbax SB-C18 narrow-bone column (2.1 × 150 mm, d_p_ = 3.5 μm). The chromatographic and MS parameters were applied as previously described by Maciejewska-Turska, M., & Zgórka, G. [49]. Data acquisition was performed using MassHunter Qualitative Navigator B.08.00 software. Compounds of interest were tentatively identified based on retention time, UV spectra, and self-analysis of their potential fragmentation patterns, supported by the comparison of obtained MS/MS spectra with records found in the literature or publicly available databases like Metlin (https://metlin.scripps.edu) [87] and HMDB (https://hmdb.ca) [37]. The identification of mangiferin isomers was performed based on the fragmentation pattern of the mangiferin reference standard.

### 4.4. Antimicrobial Evaluation

The antimicrobial activity of the *H. semenowii* extracts was tested using the microbroth dilution method according to the European Committee on Antimicrobial Susceptibility Testing [88], against a panel of reference microorganisms. The method involves determining the minimum inhibitory concentration (MIC), minimum bactericidal concentration (MBC), and minimum fungicidal concentration (MFC) of the extracts and categorizing their action as bactericidal/fungicidal (MBC/MIC ratio or MFC/MIC ratio ≤ 4) or bacteriostatic/fungistatic (MBC/MIC ratio or MFC/MIC ratio > 4). The panel of microorganisms consisted of the reference strains from the American Type Culture Collection (ATCC): *Staphylococcus aureus* ATCC 29213 (MSSA; methicillin-susceptible *S. aureus*), *S. epidermidis* ATCC 12228, *Enterococcus faecalis* ATCC 29212, *Bacillus cereus* ATCC 10876, *Escherichia coli* ATCC 25922, and *Pseudomonas aeruginosa* ATCC 27853 and fungi (yeasts) *Candida albicans* ATCC 10231 and *C. glabrata* ATCC 90030. Reference strains were selected to encompass clinically relevant bacteria and yeasts that reflect the broad diversity of pathogens encountered in healthcare settings. Gram-positive representatives included *S. aureus*, chosen for its virulence factor production and frequent involvement in skin infections, *S. epidermidis*, notable for biofilm-associated pathogenicity, *E. faecalis*, selected owing to intrinsic antibiotic resistance, and *B. cereus*, recognized for its endospore formation and role in foodborne disease. Gram-negative bacteria were represented by *E. coli* (Enterobacteriaceae), chosen for its clinical significance, and *Pseudomonas* spp., chosen for its high intrinsic resistance to antimicrobials. *C. albicans* and *C. glabrata* were included for their prevalence in human candidiasis and distinct antifungal resistance profiles. This strain selection ensured comprehensive and clinically relevant assessment of antimicrobial activity. The detailed methodology of the assay was outlined in detail in prior studies [89]. All experiments were carried out in triplicate, and the values are presented as the mode.

Statistical analysis was conducted to determine whether certain extract types exhibit different activity towards tested reference microorganisms. The analysis was conducted using a non-parametric Kruskal–Wallis test (significance set as *p* < 0.05) followed by a post hoc Dunn’s multiple comparisons test (GraphPad Prism version 6.0 for Windows; GraphPad Software, Boston, MA, USA).

### 4.5. DPPH^•^ Radical Scavenging Assay

The antioxidant activity of *H. semenowii* extracts was determined using the 1,1-diphenyl-2-picrylhydrazyl (DPPH^•^) radical scavenging method according to the methodology described in prior studies [89]. An initial test solution was prepared by dissolving 20 μL of extract in 180 μL of methanol, followed by a series of dilutions in the same solvent at concentrations ranging from 0.16 to 10 mg/mL. Each dilution was then combined with a methanol solution of DPPH^•^ in a 96-well plate, which was incubated in the dark at room temperature for 30 min. For each extract, the concentration that scavenged 50% of the initial DPPH^•^ radicals (EC_50_) and antioxidant activity index (AAI) were calculated based on absorbance measurements at 515 nm using a Biotek Epoch Microplate Spectrophotometer (Winooski, VT, USA; Software Version 3.08.01). AAI values were then compared to the criteria for assessment of antioxidant activity proposed by Sherer and Godoy [90] according to which AAI < 0.5—weak antioxidant activity, 0.5 < AAI < 1.0—moderate activity, 1.0 < AAI < 2.0—strong activity, AAI > 2.0—very strong activity. The experiments were conducted three times, and the results were reported as mean values with standard deviation (±SD).

## 5. Conclusions

The chemical composition of *Hedysarum semenowii* extracts was investigated by thorough comprehensive phytochemical analysis using HPLC/PDA and ESI-QToF/MS-MS. A total of 53 compounds were identified in extracts from both the aerial and underground parts of the plant, with flavonoids representing the most abundant class of bioactive substances. Their distribution was organ-specific—flavones predominated in the aboveground parts, while isoflavones were mainly concentrated in the roots. Flavones and isoflavones were the most representative polyphenols, highlighting their potential as chemotaxonomic indicators. Furthermore, xanthones (e.g., mangiferin and its derivatives) were found in extracts from the entire *H. semenowii* plant, including both roots and aerial parts, for the first time.

Preliminary evaluation of the antioxidant and antibacterial activities of *H. semenowii* extracts revealed that the aerial parts exhibited higher activity compared to the roots, which correlates with differences in their phytochemical composition. However, the presence of isoflavonoids and pterocarpans in *H. semenowii* roots suggests potential for further studies on biological activities related to their phytoestrogenic properties.

The results of this study provide comprehensive information on the phytochemical composition of *H. semenowii,* which may help distinguish this species from others and demonstrate its potential as a source of diverse pharmacologically active substances, depending on the plant part. Moreover, they emphasize the significance of further biological analyses and future potential pharmaceutical and medical applications.

## Figures and Tables

**Table 1:** Extracts prepared for phytochemical analysis of *H. semenowii*.

Part of the Plant	Extraction Solvent	Designated Abbreviation
Leaves	Methanol 99.8%	Hs_M99
	Methanol 70%	Hs_M70
	Methanol 50%	Hs_M50
	Ethanol 50% (maceration)	Hs_Et50M
	Ethanol 50% (ultrasonification)	Hs_Et50U
Roots	Methanol 99.8%	HsR_M99
	Methanol 70%	HsR_M70
	Methanol 50%	HsR_M50

**Table 3:** The content of mangiferin and isomangiferin in the extracts from *H. semenowii* leaves.

Compound	Extract Number * [mg/g Extract] and (SD; RSD)				
	Hs_M99	Hs_M70	Hs_M50	Hs_Et50M	Hs_Et50U
mangiferin	1.52 (0.018; 1.235)	1.89 0.062; 3.313)	2.00 (0.005; 0.259)	4.12 (0.017; 0.425)	5.46 (0.014; 0.256)
isomangiferin	0.19 (0.004; 2.663)	0.10 (0.003; 3.331)	0.18 (0.002; 11.298)	0.59 (0.024; 4.178)	0.61 (0.004; 0.789)

* Hs_M99—*H. semenowii* leaf extract in methanol 99.8%, Hs_M70—*H. semenowii* leaf extract in methanol 70%, Hs_M50—*H. semenowii* leaf extract in methanol 50%, Hs_Et50M—*H. semenowii* leaf extract in ethanol 50% (maceration), Hs_Et50U—*H. semenowii* leaf extract in ethanol 50%50% (ultrasonification), HsR_M99—*H. semenowii* root extract in methanol 99.8%, HsR_M70—*H. semenowii* root extract in methanol 70%, HsR_M50—*H. semenowii* root extract in methanol 50%.

**Table 4:** Antibacterial and antifungal activity of the extracts from *H. semenowii* (mg/mL) leaves.

Reference Microorganism		Hs_M99			Hs_M70			Hs_M50			Hs_Et50M			Hs_Et50U		
		MIC	MBC	MBC/MIC	MIC	MBC	MBC/MIC	MIC	MBC	MBC/MIC	MIC	MBC	MBC/MIC	MIC	MBC	MBC/MIC
*G*(+)	*Staphylococcus epidermidis* ATCC 12228	0.5	1	2	1	2	2	1	2	2	0.5	1	2	0.5	1	2
	*Staphylococcus aureus* ATCC 29213	0.5	1	2	4	4	1	2	4	2	1	2	2	1	2	2
	*Enterococcus faecalis* ATCC 29212	2	8	4	16	16	1	16	16	1	2	4	2	2	8	4
	*Bacillus cereus* ATCC 10876	2	2	1	4	4	1	4	4	1	1	2	2	2	4	2
*G*(−)	*Escherichia coli* ATCC 25922	8	8	1	8	8	1	8	8	1	4	4	1	8	8	1
	*Pseudomonas aeruginosa* ATCC 27853	2	4	2	2	4	2	2	2	1	1	2	1	1	2	2
		**MIC**	**MFC**	**MFC/MIC**	**MIC**	**MFC**	**MFC/MIC**	**MIC**	**MFC**	**MFC/MIC**	**MIC**	**MFC**	**MFC/MIC**	**MIC**	**MFC**	**MFC/MIC**
*Y*	*Candida albicans* ATCC 10231	4	8	2	8	8	1	4	8	2	8	8	1	4	8	2
	*Candida glabrata* ATCC 90030	8	16	2	8	16	2	4	8	2	8	16	2	8	8	1

G(+)—Gram-positive bacteria; G(−)—Gram-negative bacteria; Y—yeast (fungi); MIC—minimum inhibitory concentration, MBC—minimum bactericidal concentration, MFC—minimum fungicidal concentration. Hs_M99—*H. semenowii* leaf extract in methanol 99.8%, Hs_M70—*H. semenowii* leaf extract in methanol 70%, Hs_M50—*H. semenowii* leaf extract in methanol 50%, Hs_Et50M—*H. semenowii* leaf extract in ethanol 50% (maceration), Hs_Et50U—*H. semenowii* leaf extract in ethanol 50%50% (ultrasonification), HsR_M99—*H. semenowii* root extract in methanol 99.8%, HsR_M70—*H. semenowii* root extract in methanol 70%, HsR_M50—*H. semenowii* root extract in methanol 50%.

**Table 5:** Antibacterial and antifungal activity of the extracts from *H. semenowii* (mg/mL) roots.

Reference Microorganism		HsR_M99			HsR_M70			HsR_M50		
		MIC	MBC	MBC/MIC	MIC	MBC	MBC/MIC	MIC	MBC	MBC/MIC
*G*(+)	*Staphylococcus epidermidis* ATCC 12228	1	4	4	2	8	2	4	8	2
	*Staphylococcus aureus* ATCC 29213	4	8	2	8	8	1	16	16	1
	*Enterococcus faecalis* ATCC 29212	16	>16	nd	8	>16	nd	16	>16	nd
	*Bacillus cereus* ATCC 10876	4	4	1	4	8	2	8	8	1
*G*(−)	*Escherichia coli* ATCC 25922	16	16	1	16	16	1	16	16	1
	*Pseudomonas aeruginosa* ATCC 27853	8	8	1	8	8	1	8	8	1
		**MIC**	**MFC**	**MFC/MIC**	**MIC**	**MFC**	**MFC/MIC**	**MIC**	**MFC**	**MFC/MIC**
*Y*	*Candida albicans* ATCC 10231	4	8	2	4	8	2	4	8	2
	*Candida glabrata* ATCC 90030	8	16	2	8	16	2	8	16	2

G(+)—Gram-positive bacteria; G(−)—Gram-negative bacteria; Y—yeast (fungi); MIC—minimum inhibitory concentration, MBC—minimum bactericidal concentration, MFC—minimum fungicidal concentration. Hs_M99—*H. semenowii* leaf extract in methanol 99.8%, Hs_M70—*H. semenowii* leaf extract in methanol 70%, Hs_M50—*H. semenowii* leaf extract in methanol 50%, Hs_Et50M—*H. semenowii* leaf extract in ethanol 50% (maceration), Hs_Et50U—*H. semenowii* leaf extract in ethanol 50%50% (ultrasonification), HsR_M99—*H. semenowii* root extract in methanol 99.8%, HsR_M70—*H. semenowii* root extract in methanol 70%, HsR_M50—*H. semenowii* root extract in methanol 50%, nd—not determined.

**Table 6:** Results of Kruskal–Wallis test and post hoc Dunn’s multiple-comparisons test, showing significance of differences in antibacterial activity of *Hedysarum semenowii* extracts.

Reference Strains	Kruskal–Wallis Test		Dunn’s Multiple-Comparisons Test
	H (7)	*p*	
*S. epidermidis* ATCC 12228	18.01	0.012	nd
*S. aureus* ATCC 29213	21.63	0.003	Hs_M99 vs. HsR_M50
*E. faecalis* ATCC 29212	20.08	0.005	nd
*B. cereus* ATCC 10879	18.27	0.011	Hs_Et50M vs. HsR_M50
*E. coli* ATCC 25922	20.18	0.005	nd
*P. aeruginosa* ATCC 27853	19.68	0.006	nd
*C. albicans* ATCC 10231	12.99	0.072	-
*C. glabrata* ATCC 90030	10.43	0.165	-

H (7)—test value, *p*—significance level, nd—not determined.

**Table 7:** Antioxidant activity of the extracts from *H. semenowii* leaves.

	Hs_M99	Hs_M70	Hs_M50	Hs_Et50M	Hs_Et50U
EC_50_, µg/mL	48.3 ± 6.27	84.6 ± 8.4	206.6 ± 30.4	36.7 ± 2.35	50.9 ± 8.81
AAI	1.6 ± 0.2	0.90 ± 0.09	0.37 ± 0.05	2.07 ± 0.13	1.49 ± 0.26

Hs_M99—*H. semenowii* leaf extract in methanol 99.8%, Hs_M70—*H. semenowii* leaf extract in methanol 70%, Hs_M50—*H. semenowii* leaf extract in methanol 50%, Hs_Et50M—*H. semenowii* leaf extract in ethanol 50% (maceration), Hs_Et50U—*H. semenowii* leaf extract in ethanol 50%50% (ultrasonification), HsR_M99—*H. semenowii* root extract in methanol 99.8%, HsR_M70—*H. semenowii* root extract in methanol 70%, HsR_M50—*H. semenowii* root extract in methanol 50%.

**Table 8:** RP-LC/PDA method validation parameters.

Reference Substance	Regression Equation	*R* ^2^	Linearity Range (mg/mL)	LOD (μg/mL)	LOQ (μg/mL)	Intraday Precision (*n* = 3) (%)	Interday Precision (*n =* 3) (%)
mangiferin	y = 4 × 10^7^x + 128,265	0.9993	0.0125–0.2	1.042	3.125	1.32	0.89

## Data Availability

The original contributions presented in this study are included in the article/Supplementary Materials. Further inquiries can be directed to the corresponding authors.

## Supplementary Materials

The following supporting information can be downloaded at https://www.mdpi.com/article/10.3390/molecules30234503/s1, Figure S1: RP-LC/PDA chromatogram of major compounds detected at λ = 254 nm in *Hedysarum semenowii* extracts.
