# Supplementary material for: Phytochemical Composition and Antimicrobial and Antioxidant Activity of Hedysarum semenowii (Fabaceae)
Source: Molecules. 2025 Nov 21;30(23):4503. doi: 10.3390/molecules30234503 (PMC12693121; doi:10.3390/molecules30234503)
Supplement: Supplementary file 1 [file molecules-30-04503-s001.zip › molecules-3931735-supplementary.pdf]

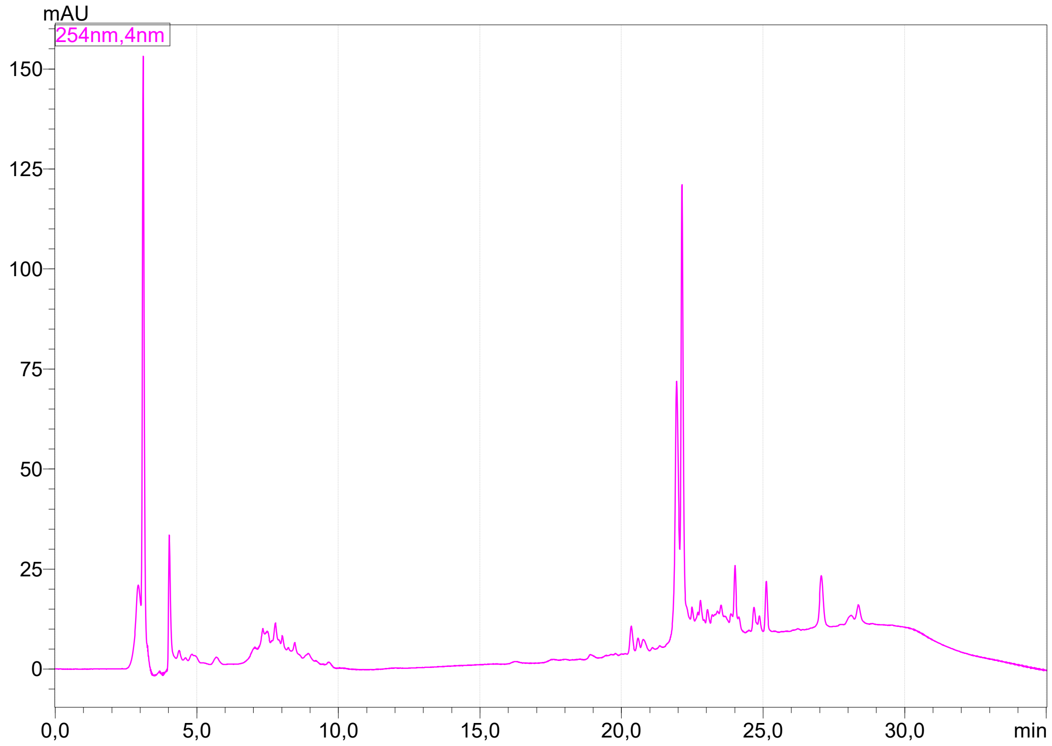
mAU
254nm,4nm
150
125
100
75
50
25
0
0,0
5,0
10,0
15,0
20,0
25,0
30,0
min

(a)

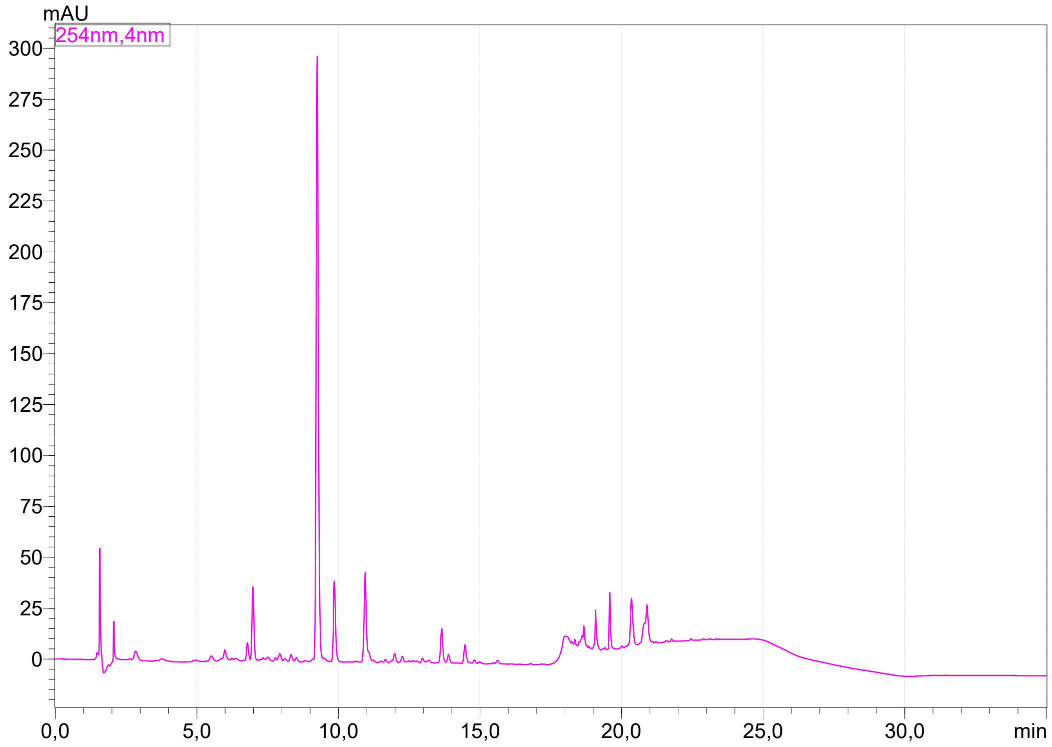
mAU
254nm,4nm
300
275
250
225
200
175
150
125
100
75
50
25
0
0,0
5,0
10,0
15,0
20,0
25,0
30,0
min

(b)

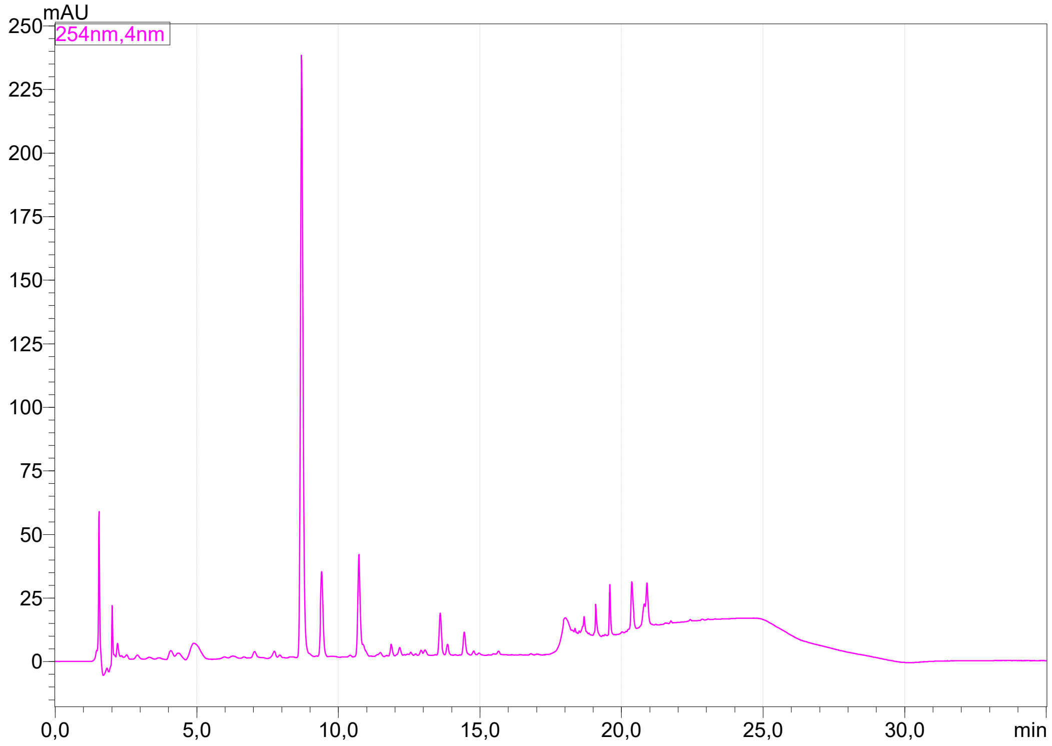

(c)

Figure S1. RP-LC/PDA chromatogram of major compounds detected at λ=254 nm in *Hedysarum semenowii* extracts. (a) RP-LC/PDA chromatogram of extract HsR_M70; (b) RP-LC/PDA chromatogram of extract Hs_M70; (c) RP-LC/PDA chromatogram of extract Hs_Et50U.
